# Preconditioning Triggered by Carbon Monoxide (CO) Provides Neuronal Protection Following Perinatal Hypoxia-Ischemia

**DOI:** 10.1371/journal.pone.0042632

**Published:** 2012-08-28

**Authors:** Cláudia S. F. Queiroga, Simone Tomasi, Marius Widerøe, Paula M. Alves, Alessandro Vercelli, Helena L. A. Vieira

**Affiliations:** 1 Chronic Diseases Research Center (CEDOC), Faculdade de Ciências Médicas, Universidade Nova de Lisboa, Lisboa, Portugal; 2 Instituto de Biologia Experimental e Tecnológica (IBET)/Instituto de Tecnologia Química e Biológica (ITQB), Universidade Nova de Lisboa, Oeiras, Portugal; 3 Neuroscience Institute Cavalieri Ottolenghi (NICO) - AOU San Luigi Gonzaga, Orbassano, Turin, Italy; 4 Department of Anatomy, Pharmacology and Forensic Medicine, University of Turin Medical School, Turin, Italy; 5 Department of Laboratory Medicine, Children's and Women's Health, Norwegian University of Science and Technology, Trondheim, Norway; University of South Florida, United States of America

## Abstract

Perinatal hypoxia-ischemia is a major cause of acute mortality in newborns and cognitive and motor impairments in children. Cerebral hypoxia-ischemia leads to excitotoxicity and necrotic and apoptotic cell death, in which mitochondria play a major role. Increased resistance against major damage can be achieved by preconditioning triggered by subtle insults. CO, a toxic molecule that is also generated endogenously, may have a role in preconditioning as low doses can protect against inflammation and apoptosis. In this study, the role of CO-induced preconditioning on neurons was addressed *in vitro* and *in vivo*. The effect of 1 h of CO treatment on neuronal death (plasmatic membrane permeabilization and chromatin condensation) and bcl-2 expression was studied in cerebellar granule cells undergoing to glutamate-induced apoptosis. CO's role was studied *in vivo* in the Rice-Vannucci model of neonatal hypoxia-ischemia (common carotid artery ligature +75 min at 8% oxygen). Apoptotic cells, assessed by Nissl staining were counted with a stereological approach and cleaved caspase 3-positive profiles in the hippocampus were assessed. Apoptotic hallmarks were analyzed in hippocampal extracts by Western Blot. CO inhibited excitotoxicity-induced cell death and increased Bcl-2 mRNA in primary cultures of neurons. *In vivo*, CO prevented hypoxia-ischemia induced apoptosis in the hippocampus, limited cytochrome c released from mitochondria and reduced activation of caspase-3. Still, Bcl-2 protein levels were higher in hippocampus of CO pre-treated rat pups. Our results show that CO preconditioning elicits a molecular cascade that limits neuronal apoptosis. This could represent an innovative therapeutic strategy for high-risk cerebral hypoxia-ischemia patients, in particular neonates.

## Introduction

Perinatal hypoxia-ischemia (HI) remains a major cause of acute mortality in newborns and of cognitive and motor impairments in children [1]. Cerebral damage results from oxygen and tissue energy depletion that lead to: acidosis, inflammation, glutamate excitotoxicity, cell death and generation of reactive oxygen species (ROS) during reperfusion [2]. Cerebral hypoxia-ischemia induces distinct types of cell death: within the ischemic *core*, rapid cell death occurs mainly as necrosis; while in the *penumbra*, the region around the ischemic core, delayed apoptotic cell death takes place hours and days after the insult, contributing to secondary damage [3]. In the developing brain, apoptosis also plays a homeostatic role, and many pro-apoptotic factors are normally up-regulated during early stages of maturation [4], [5]. Therefore, apoptosis is closely related to the injury response after hypoxia-ischemia in the immature brain of newborn infants [6]. Mitochondria play a major role in death of mammalian cells [7]. During the apoptotic process and upon mitochondrial membrane permeability, several biochemical molecules confined to the inter-membrane space are released to the cytosol thus activating proteases and nucleases. For example, cytochrome c released from mitochondria interacts with apoptotic protease activating factor 1 (Apaf-1) and caspase-9 to form the apoptosome that activates caspase-3 leading to cell death [8].

Currently, hypothermia is the only treatment used clinically for minimizing cerebral damage after perinatal hypoxia-ischemia, but it has limited efficiency and its use has also limitations [9]. Other therapies, which either can be used alone or in combination with hypothermia, are therefore needed. Preconditioning (PC) induction consists of an insult that does not cause damage, but triggers a protective state (tolerance) that increase cellular resistance against a subsequent and more severe challenge [10], [11]. PC can induce an *early* response (minutes or hours) or a *late* response within days including *de novo* protein synthesis [12]. Furthermore, clinical studies with patients suffering from transient ischemic attacks (TIA) [13] and animal models [12], [14], [15] have suggested that cerebral tolerance induced by a PC state is an efficient strategy to protect brain tissue against HI. Thus, preconditioning processes are promising alternatives for therapy in patients at high risk of suffering HI. Indeed, perinatal HI may eventually be predicted based on known risk factors associated with previous ischemic episodes including intrauterine fetal distress and hypoxic-ischemic insults during birth [16]; Bonifacio et al. 2011). Also, PC-based therapies could be useful for neonates going through major heart surgery with associated risks of global cerebral ischemia [10], [13], [17].

Carbon monoxide (CO) is commonly known to be toxic. This is due to its high affinity for haem-proteins, which can compromise oxygen delivery to tissues (carboxy-haemglobin) or can decrease oxidative phosphorylation at the cellular level by binding to cytochrome c oxidase [18]. CO is an endogenous molecule generated by haem-oxygenase (HO) activity along with the production of free iron and biliverdin [19]. Low doses of exogenous CO are cytoprotective against inflammation and apoptosis, in particular following cardiovascular incidents, organ rejection and autoimmune disease in several models [19]. Also, in rat retinal ganglion cells, inhalation of 250 ppm of CO protected against ischemia-reperfusion injury [20]. In the central nervous system (CNS), low amounts of CO limit neuroinflammation in a model of multiple sclerosis [21] and induced vasodilation, presenting cytoprotective effects in the cerebral circulation in a model of epileptic seizures in newborn piglets [22]. CO treatment also decreased infarct volume and brain damage in adult models of transient and permanent focal cerebral ischemia when the animals were exposed to CO immediately after middle cerebral artery occlusion [23], [24]. Nevertheless, the cellular mechanisms involved in CO-induced neuroprotection are still not fully understood. In primary cultures of cerebellar neurons, CO triggers preconditioning and prevents apoptosis by ROS signaling and modulation of soluble guanylyl cyclase, nitric oxide synthase and mitochondrial ATP dependent potassium channel [25]. Likewise, in primary cultures of astrocytes, CO inhibits apoptosis by directly targeting mitochondria and preventing their membrane permeabilization, which is also dependent on ROS and protein glutathionylation signaling [26].

Since preconditioning emerges as a promising strategy to limit brain damage following perinatal ischemia, we have examined the ability of CO to induce preconditioning and to limit apoptosis in the hippocampus in the present study. Pre-treatment of rat pups with CO prevented hippocampal cell death *via*: an increase on Bcl-2 expression, a decrease on cytochrome c translocation from mitochondria into cytosol and an inhibition of caspase-3 activation. To our knowledge, this is the first study to use CO preconditioning to prevent hypoxia-ischemia-induced neuronal death in the developing brain.

## Materials and Methods

### *In vitro* experiments

#### Materials

All chemicals were purchased from Sigma-Aldrich (Munich, Germany) unless stated otherwise. Plastic tissue culture dishes were from Nunc (Roskilde, Denmark); fetal bovine serum (FBS), glutamine, penicillin-streptomycin solution and Dulbecco's Minimum Essential Medium (DMEM) were obtained from Gibco (Paisley, UK). CO 100% was purchased as compressed gas (Linde, Germany).

#### Cell Cultures

Primary cerebellar granule cells were prepared as described by Schousboe [27] from Wistar rats purchased from Instituto de Higiene e Medicina Tropical (Lisboa, Portugal). Briefly, cells were isolated from postnatal day 7(P7) rat cerebella, after mild trypsinization followed by trituration in a DNase solution containing soybean trypsin inhibitor. Cells were suspended (1×10^6^ cells/mL) and cultured in BME basal medium containing 12 mM glucose, 7.3 µM p-Aminobenzoic acid, 4 µg/L insulin, 2 mM glutamine, 1% (vol/vol) penicillin-streptomycin solution and 10% (vol/vol) FBS. Cells were cultured in 24- and 96-well poly-D-lysine coated plates and maintained in humidified atmosphere of 7% CO_2_ at 37°C. To prevent glia proliferation, cytosine arabinoside (20 µM) was added 48 h after seeding. The experiments were performed on 1-week-old cerebellar granule neuronal culture. All experiments were performed at least in triplicate.

#### Preparation of CO solutions

Fresh stock solutions of CO gas were prepared each day and sealed. Phosphate-buffered saline (PBS) was saturated by bubbling 100% of CO gas for 30 minutes to produce a 10^−3^ M stock solution. The concentration of CO in solution was determined spectrophotometrically by measuring the conversion of deoxymyoglobin to carbon monoxymyoglobin, as described by Motterlini (Motterlini 2002).

#### Cell treatments, induction of apoptosis and assessment of apoptosis-associated parameters

Neuronal cells were cultured on poly-D-lysine-coated coverslips. Neuronal apoptosis was induced immediately after the end of CO exposure with 10 to 30 µM of glutamate over a 24 h period to mimic excitotoxicity, which is a consequence of cerebral ischemia [25]. For inhibition of cell death, cerebellar granule cells were treated with 10 µM CO for 1 h prior to glutamate addition. Neurons were stained with Hoechst 33342 (2 µM, Sigma) and Propidium Iodide (PI, 1 µM, Molecular Probes, USA) followed by quantitative assessment of chromatin condensation and cell viability, respectively. Cells were observed on a Leica DMRB microscope using a filter with a bandpass of 340–380 nm (UV).

#### Real Time Quantitative PCR

After 6 or 24 h of CO treatment, RNA was extracted from primary cerebellar neurons (3×10^6^ cells) using the High Pure RNA isolation Kit (Roche, Germany); cDNA was synthesized from RNA (Transcriptor High Fidelity cDNA Synthesis Kit, Roche, Germany). For real time quantitative PCR (RT-qPCR), forward and reverse primer sequences specific for bcl-2 gene consisted of 5′-GGTGGAGGAACTCTTCAGGG-3′ and 5′-GAGACAGCCAGGAGAAATCA-3′, respectively. An internal control used expression of cyclophilin A, a constitutive protein, using forward and reverse sequences: 5′-ATGGCAAATGCTGGACCAAA-3′ and 5′-GCCTTCTTTCACCTTCCCAAA-3′. The RT-qPCRwas performed according to manufacturer indications (LightCycler® FastStart DNA Master^PLUS^ SYBR Green I, Roche Diagnostics, Germany). Amplification used the following protocol: denaturation at 95°C for 10 minutes; amplification for 35 cycles, at 95°C for 15 seconds, 60°C for 6 seconds, 72°C for 15 seconds with a single fluorescent measurement; 95°C for 15 seconds, 60°C for 15 seconds, 95°C for 15 seconds with a continuous fluorescence measurement for the melting curve; finally the cooling step was at 40°C. The results were expressed in percentage relative to the control sample.

### *In vivo* experiments

#### Animals

P4–P7 (postnatal day 4 to postnatal day 7) Wistar rat pups (n = 74, Janvier, France) weighing on average 27 (±1,4) and 35 g (±1,5) respectively were used. Great differences in pup's weight are due to the fast rate of growing at this period of life. All animal experimental procedures were carried out in strict accordance with the recommendations in the European Community Council Directive 86/609/EEC (November 24, 1986), authorization 17/2010-B (June 30, 2010) by Italian Ministry of Health and University of Turin institutional guidelines on animal welfare (law 116/92 on Care and Protection of Living Animals Undergoing Experimental or Other Scientific Procedures). The protocol was approved by Turin University Bioethical Committee. Animals were given *ad libitum* access to food and water and kept on 12h:12h light/dark cycle. Pup's sex was not taken into account, and male and female pups were used randomly for introducing variability in the study and for being more representative of relevant patient populations. All efforts were made to minimize suffering and limit the number of animals used.

#### Experimental plan

**Figure 1:**
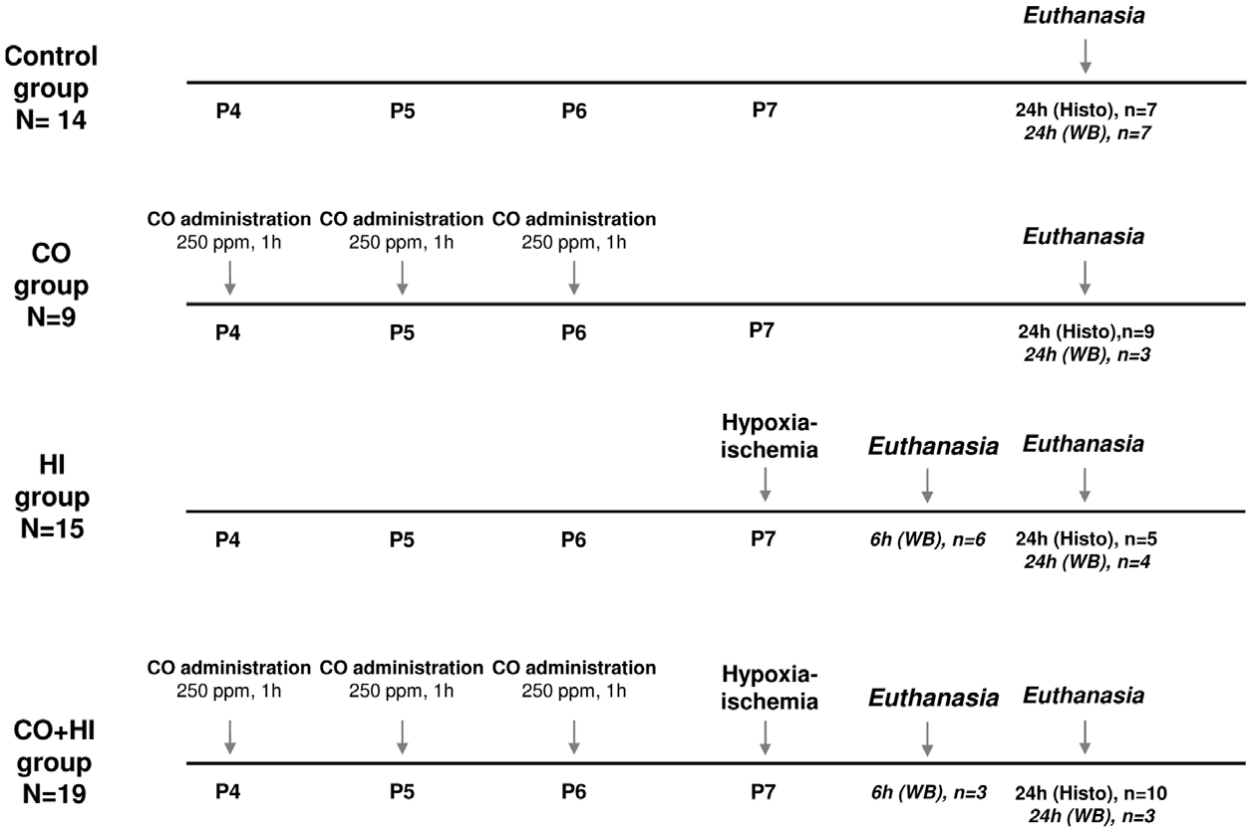
Experimental groups and time-points schematic representation. **Control group**, *n = 22*, untreated animals that did not suffer any treatment; **Carbon Monoxide (CO) group**, *n = 16*, subjected to 3 exposures of 250 ppm, for 1 h at P4, P5 and P6; **Hypoxia-Ischemia (HI) group**, *n = 17*, animals that underwent surgery and hypoxia (8% of O_2_ in nitrogen) exposure for 75 minutes; **CO+HI group**, *n = 19*, CO treatment plus hypoxia-ischemia. Animals were euthanized at 6 and 24 h *post*-HI. Brains were collected and analyzed for lesion volume and cell death markers, as described in the methods section. *Histo*, for brains analyzed by histological methods; *WB*, for brains collected and processed for western blot analysis.

To test whether CO prevented HI-induced neurodegeneration, and to ensure that CO administration did not cause toxicity, animals were randomly assigned to four experimental groups: CONTROL group (sham surgery without hypoxia exposure, *n = 22*), CO+SHAM group (CO exposure prior to sham surgery without hypoxia exposure, *n = 16*), HI group (hypoxia-ischemia, *n = 17*) and CO+HI group (CO exposure prior to HI, *n = 19*) (Fig. 1). Animals were randomly assigned to each group through computer-generated (Excel) randomization schedules before performing any experimental procedure. During the HI procedure and CO exposure pups in all experiment groups were kept separate from their mother the equal period of time. Rats were sacrificed 6 and 24 hours after HI onset. All subsequent procedures were done by researchers who were blinded to the experimental groups.

#### Carbon monoxide exposure

Because this is the first study of CO as neuroprotective agent against perinatal ischemia, using rat pups, CO exposure conditions (dose and time) were extrapolated from several previous *in vivo* studies for liver [28], [29], heart [30] or adult brain [24]. To induce a preconditioning state and test the neuroprotective role of CO, rat pups were exposed to 250 ppm of CO for 1 h at P4, P5 and P6. During the exposure, gas concentration was carefully monitored by a CO analyser (Interscan, Chatsworth, US) and temperature was kept at 37°C. At the end of each CO administration, rat pups were returned to their cages. During CO exposure time, control pups (without CO exposure) were also kept separated from their mothers.

#### Model of neonatal cerebral hypoxia-ischemia

HI was induced in P7 rat pups, according to the Rice-Vannucci modification of the Levine procedure [31], [32]. The P7 rat was chosen because its level of cortical development closely resembles that of 32–34 week gestation: cortical layering is complete and low grade myelination is visible [33]. Rat pups were anesthetized with isoflurane (Isoflurane Vet, Alcyon Italia, Marene, Italy, 4% during induction, then maintained with 2.5%), presented in a mixture of 30∶70 O_2_/N_2_O, delivered with a face-mask throughout the surgery. Through a mid-neck incision, the left common carotid artery (CCA) was exposed and double-ligated by means of 4/0 silk suture in order to permanently interrupt the blood flow. Care was taken to avoid damage to the adjacent vagal nerve. The procedure was completed in 10–15 min. After 2 h of recovery and feeding, pups were exposed to humidified 8% O_2_–92% N_2_ gas mixture [34] for 75 minutes in a home-made acrylic hypoxic chamber (2,7 L). During all surgical procedures and exposure in the hypoxic chamber the body temperature of the pups was maintained with a heating carpet. Pups were allowed to recover for 5 min in room air, before returned to their mothers. Sham-operated animals underwent the same surgical procedure without CCA ligation and put into a similar chamber for the same period of time as the HI group to mimic the time away from the mother. The temperature was monitored and maintained at 37°C throughout all procedure. Cell death assessment was performed at 6 h and 24 h after HI, based on our *in vitro* experimental data (data not shown, [25], [26], [35].

#### Tissue processing

For histological and immunohistochemical staining, animals were deeply anesthetized by intraperitoneal injection of chloral hydrate 24 h after surgery and transcardially perfused with PBS, followed by buffered paraformaldehyde (4% in 0.1 M phosphate buffer, PB, pH 7.4). Brains were post-fixed overnight in the same solution, infiltrated with 30% sucrose in 0.1 M PB for cryoprotection, frozen, and stored at −20°C. 50 µm-thick serial coronal sections were cut on the cryostat; every sixth slice was mounted on gelatine-coated slides for histological and immunological staining. For Western blot assays, animals were decapitated and brains dissected in ice-cold water, kept in ice for 15 min, and then frozen at −80°C.

#### Histological and immunohistochemical assessment

To define ischemic boundaries and precisely identify apoptotic profiles, serial sections were stained with cresyl violet. For immunohistochemistry, sections were immersed in 10% normal donkey serum (NDS), then incubated overnight in antibodies against cleaved-caspase 3 (1∶200, Cell Signaling Technology, Beverly, MA, US), S100β (1∶1000, Sigma-Aldrich) or Ki67 (1∶400, Novocastra, Newcastle, UK) at 4°C; primary antibodies were prepared in 10% NDS solution. For Ki67 immunohistochemistry, sections were initially heated in 0.01 M citrate buffer (pH 6.0) for 3 minutes at 800 W using a microwave oven prior to primary antibody incubation to enhance antigen retrieval. Cy2- or Cy3-conjugated anti-rabbit or anti-mouse secondary antibodies (1∶200 and 1∶400 respectively, 2 h at room temperature, Jackson ImmunoResearch Laboratories, West Grove, USA) were used to visualize binding of primary antibodies; cell nuclei were counterstained with 4′,6′-diamidino-2-phenylindole (DAPI, Sigma-Aldrich). Control tissue was processed as above with non-immune serum instead of the primary antibody. Immunoreacted sections were examined with Nikon 90i epifluorescence microscope by an investigator blinded to the experimental groups. Co-localization was determined on a Leica TCS SP5 confocal laser scanning microscope equipped with argon 488 nm, helium–neon 543 nm, and helium–neon 633 mn lasers. Double-immunolabeled cells were analyzed using three-dimensional (3D) reconstructed images with the x–z and y–z orthogonal projections, using Leica LAS AF software.

#### Stereological counts in the hippocampus

Cresyl violet-stained sections were analysed with 100× oil-immersion objective for stereological estimation of apoptotic profiles. Sections were visualized using a Nikon Eclipse E600 microscope equipped with a motorized stage controller; they were photographed with a CCD camera (OptronicsMicrofire, Goleta, CA, USA). Analyses were done by an investigator blinded to the experimental groups. We used the optical fractionator method for stereological estimates of apoptotic profiles in the hippocampal formation; the volume of hippocampus was calculated according to the Cavalieri's principle (Neurolucida 8.0 User's Guide—Document Version NL0108-08.10 Microbrightfield Inc., 1988–2008.); all analyses were done with use of StereoInvestigator (Microbrightfield, Williston, VT, USA). Series were 300 µm spaced; the first section of each series was dorsal to the hippocampal white matter; subsequent collected sections were equidistant from this reference slice; each series consisted of 9 sections. The procedure yields a series of systematically random sections (a requirement for the optical fractionator methods, Lyster et al 2005; West et al 1991). Stereological sampling was done according to Fitting (Fitting et al., 2008). An initial pilot study optimized the sampling scheme. The coefficient of sampling error (CE) was calculated to determine the appropriate number and size of disector counting frames. When considering 9 sections per case, a sampling grid of 400×400 µm, with a 25×25 µm counting frame and a disector height of 20 µm (disector volume = 12500 µm^3^) was chosen; the CE varied between 0.01 and 0.1. To avoid oversampling, two guard zones were set on top and bottom of sections, each corresponding to 10% of the focally measured section thickness. Only neurons bearing morphological changes strongly suggestive for apoptosis, namely cell shrinkage, chromatin condensation and visible apoptotic bodies were considered dying cells and counted. The estimated total (T) number of objects (i.e., apoptotic profiles) was finally calculated in accordance to the following formula, as previously described by Fitting [36]

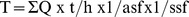
where ΣQ is the number of objects counted in the disectors, t is the section thickness, h is the height of disector probe, asf is the ratio between counting frames area and grid step along x and y axis, and ssf is the section sampling fraction and was set to 1/6. To avoid bias due to asymmetrical cutting, the total estimated number of objects was then normalized to the sampled volume and density values are presented (Fig. S1).

#### Immunoblotting

The apoptotic hall-markers: (i) Bcl-2 expression, (ii) release of cytochrome c from mitochondria and (iii) caspase activation, were assessed in hippocampus isolated 6 and 24 h after the insult. Rat pups were first anesthetized then decapitated, the hippocampus dissected and stored at −80°C until analysis; tissue was triturated manually with Potter Elvehjem in lysis buffer (15 mMTris-HCl, pH 7.6, 320 mM sucrose, 1 mM DTT, 1 mM MgCl_2_, 0.5% protease inhibitor, 3 mM EDTA-K, 30 µg/mL CsA). For total cell extract analysis, samples were sonicated before protein quantification. For mitochondrial analysis, this protein extract was centrifuged at 800 *g* for 10 min at 4°C, supernatant was re-centrifuged at 9200 *g* for 15 min at 4°C and the pellet containing mitochondria was stored for Western blot analysis. Protein content (from total extract or mitochondrial fraction) was determined by BCA assay (Pierce, Illinois). Proteins (20–40 µg) were separated on SDS-PAGE (12% polyacrylamide), transferred to PVDF membranes, blocked in 5% skim milk in Tris-buffered saline (containing 0.1% of Tween 20) for 1 h at room temperature. Equal amounts of protein were confirmed by the internal control assessment of ß-actin. The following primary antibodies were used overnight at 4°C: anti-Bcl-2 (Santa Cruz Biotechnology, 1∶5000); anti-cytochrome c (Abcam, 1∶2000); anti-active caspase-3 (Cell Signaling, 1∶5000); anti ß-actin (Santa Cruz Biotechnology, 1∶5000); all were diluted in blocking solution. Blots were developed using the ECL chemiluminescence kit (Amersham Bioscience, UK) after incubation with HRP-labeled anti-mouse or anti-rabbit IgG (GE Healthcare, UK, 1∶5000) for 1 hour at RT. The area and intensity of bands were quantified by densitometry analysis (GraphPad Prism 4), and were normalized to the positive control (100%). Each experiment was repeated three times, and gave similar results.

#### Statistical analysis of data

All experiments were carried out at least in triplicate; values are mean ± SD, n≥3. Error bars, corresponding to standard deviation. Statistical comparisons were performed using ANOVA: single factor with replication, with P<0.05, n≥3. P<0.05 means that samples are significantly different at a confidence level of 95%. All statistical comparisons were conducted using the SPSS package (version 18, SPSS Inc., Chicago, IL, USA). For *in vivo* study, data distribution and equality of variances were initially assessed by the Shapiro-Wilk [37]and the Levene median [38] tests, respectively. One-way ANOVA was applied to determine overall significant differences in the number of apoptotic cells among groups. *Post-hoc* analyses were conducted when p<0.05 by Fisher's protected least significant difference (PLSD) test.

## Results

### Carbon monoxide prevents excitotoxicity-induced cell death and induces Bcl-2 expression in primary cultures of neurons

**Figure 2:**
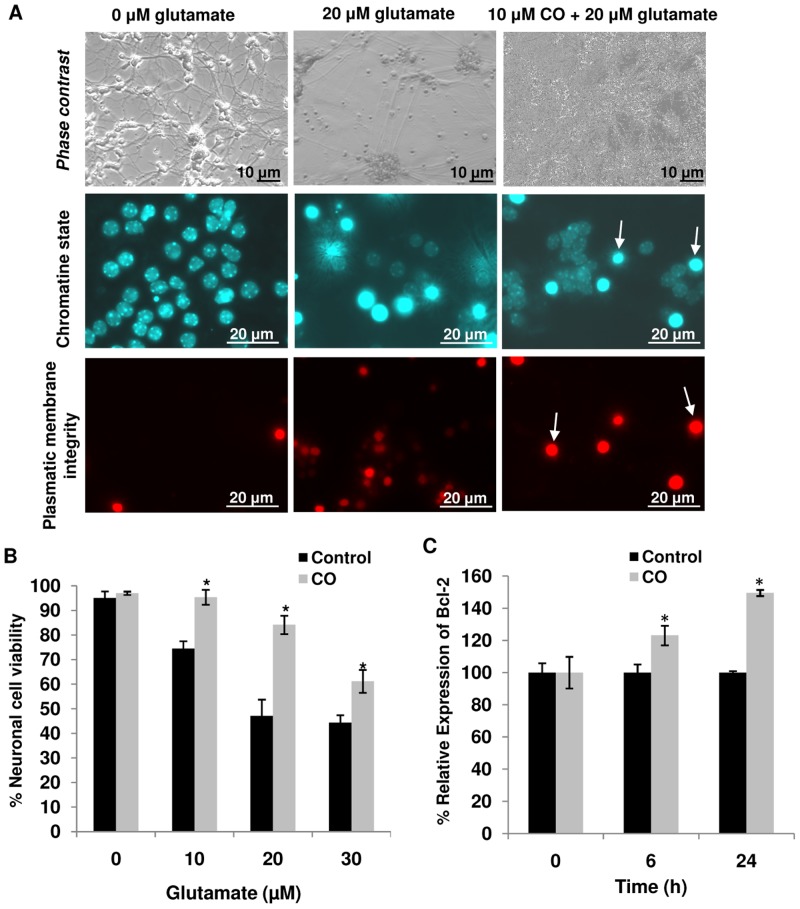
Effect of carbon monoxide treatment on neuronal apoptosis. (**A**) Representative micrographs of neurons treated or not with 20 µM of glutamate and 10 µM of CO. Apoptotic hallmarks were analyzed by fluorescent microscopy. *Upper panel*, for the photos taken with the filter for phase contrast, *middle panel*, for Hoechst (white arrows for nuclei with condensed chromatin) and *lower panel*, for propidium iodide (white arrows for cells which membrane integrity was lost). (**B**) Primary cultures of neuronal cells were pre-treated with 10 µM CO, followed by 24 h of glutamate (10–30 µM) treatment. Cell viability was assessed by counting cells containing normal nuclei and plasmatic membrane integrity. For each coverslip, at least 1500 cells were counted. All values are mean ± SD (error bars), n = 5; *p<0.05 compared to control. (**C**) The effect of 10 µM CO treatment on Bcl-2 expression was assessed by its mRNA quantification.

Primary cultures of neurons were pre-treated for 1 h with CO-saturated solution at 10 µM. After 24 h, chromatin condensation and loss of membrane integrity were assessed by fluorescence microscopy (representative photos in Fig. 2A), and CO partially prevented neuronal cell death (Fig. 2B). Additionally, Bcl-2 mRNA was measured by RT-Q-PCR, with increased expression levels at 6 h and 24 h (Fig. 2C) after CO treatment; which is in accordance with CO-related neuroprotection, since Bcl-2 is an anti-apoptotic protein.

### Effect of preconditioning on HI-induced apoptosis *in vivo*

**Figure 3:**
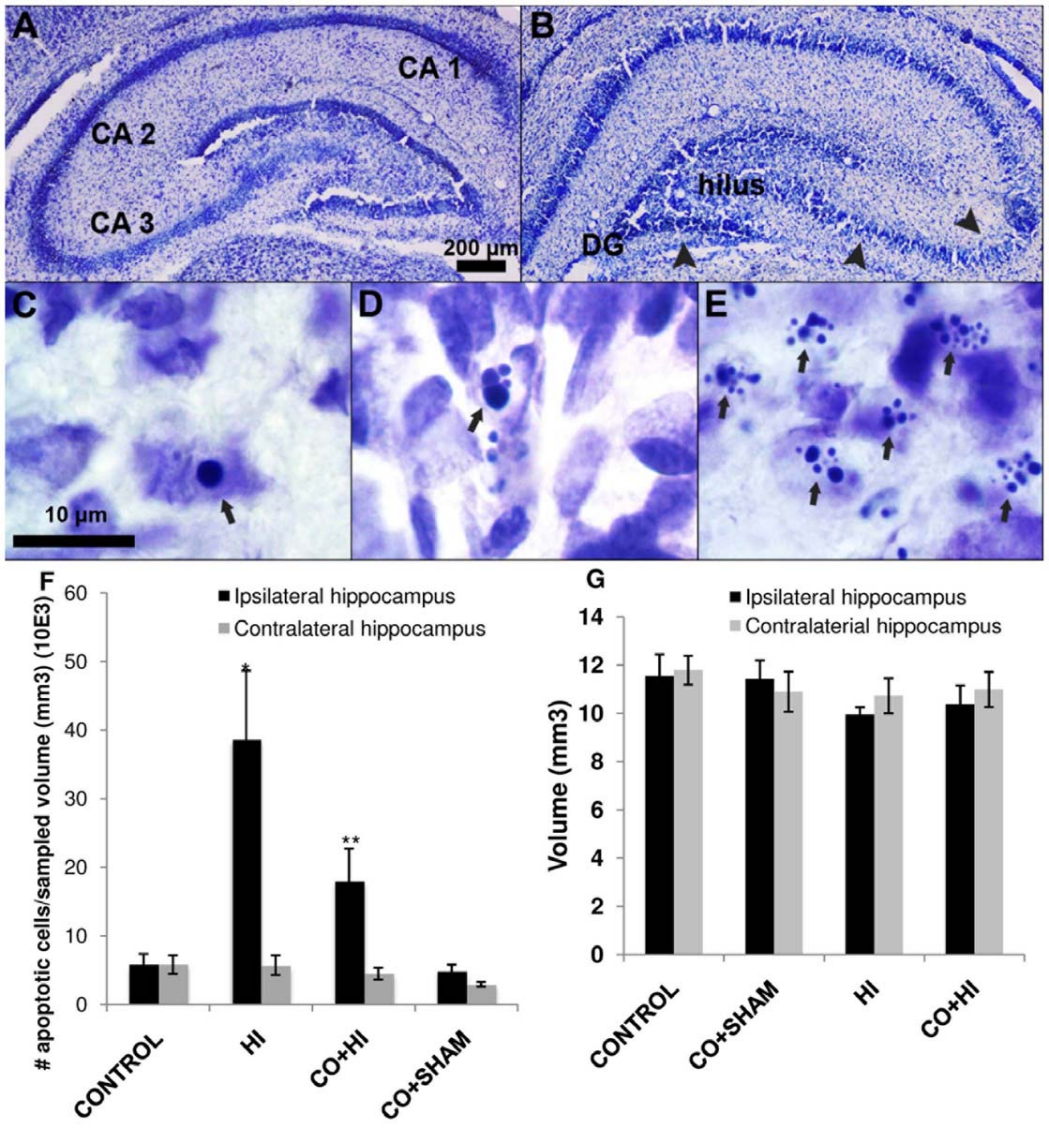
Carbon monoxide effect in hippocampus after perinatal hypoxia-ischemia – apoptotic profiles. Whereas contralateral hippocampus displayed a preserved morphology (**A**) following HI, diffuse tissue disruption was detected in the hippocampus ipsilateral to the occlusion (**B**). C–E are representative pictures of ischemic hippocampus, where diffuse apoptosis was documented; with peculiar morphological features including pyknotic nuclei (**C**), indicating early stage of apoptosis, progressive nuclear fragmentation (**D**) and karyorrhexis as confirmed by detectable apoptotic bodies (**E**). Compared to HI group, the number of apoptotic profiles was significantly lower when animals were exposed to CO prior to HI (**F**). All values are mean ± SD (error bars); *p<0.05 compared to Control group for the corresponding side and **p<0.05 compared to HI group ischemic hippocampus. (**G**) For each group there is no significant difference in cytotoxic edema volume (mm^3^) between the ipsi- and the contralateral hippocampus.

75 min of hypoxic exposure after carotid occlusion (perinatal model for cerebral hypoxia-ischemia) reliably induced mild damage in terms of cell death in the subregions of the hippocampus (Fig. 3A–E), predominantly at the level of CA2 and CA3 ipsilateral to the CCA occlusion, while the cortex did not show any detectable damage. The cytoarchitecture was partly lost due to shrinkage relative to the contralateral side, in which histological morphology was preserved (Fig. 3A–E). A great number of apoptotic profiles were detected at the level of CA1, CA2/3 and dentate gyrus (DG) after HI. Early stages of the apoptotic cascade were revealed by the presence of cells with darkly stained nuclei, condensed chromatin and dense cytoplasm (pyknosis); late stages of apoptosis were also visible in cells that had DNA fragmentation, and nuclei broken into several discrete chromatin bodies (karyorrhexis), cytoplasmic disruption, and appearance of apoptotic bodies (Fig. 3C–E). The density of apoptotic cells was significantly higher in the hippocampus ipsilateral to the occlusion (ischemic side) compared to the contralateral (intact) side, both in the HI group and the CO+HI group (Fig. 3F). However, the density of apoptotic profiles in the ischemic hemisphere was efficiently reduced (64%) in the CO+HI group compared to the HI group (Fig. 3F).

**Figure 4:**
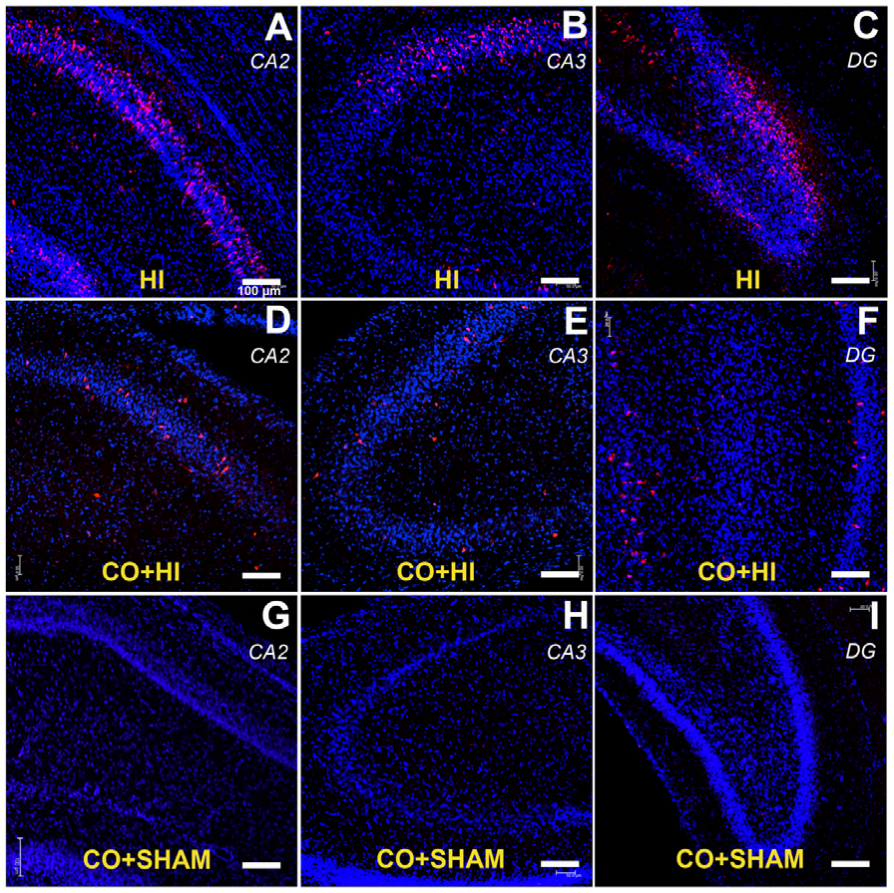
Carbon monoxide effect in hippocampus after perinatal hypoxia-ischemia – cleaved caspase 3 expression. Low (scale bar = 100 µm) magnification CLSM photographs of the hippocampus of HI (A, B), CO-HI (C, D) and CO sham operated (E, F) rat pups. In blue, DAPI-stained nuclei; in red, cleaved caspase 3-positive cells. Caspase 3-positive profiles following HI were particularly frequent in CA1–2 and in the dentate gyrus, and were decreased in number following CO preconditioning. CO preconditioning alone did not induce caspase 3 activation in sham operated animals.

Cytotoxic edema, known to contribute to early ischemic damage, may represent a confounding factor in estimating cell density and, when severe, may lead to an underestimation of apoptosis. However, this was not the case in our material: no ischemia-related differences in hippocampal volumes (mm^3^) between the hemispheres were seen in any of the groups (Fig. 3G). Similarly, the volume of ischemic or intact hippocampus did not significantly vary between groups. Accordingly, active caspase 3 positive cells were predominantly detected in CA2, CA3 and DG regions of the hippocampus in the HI group (Fig. 4A–C), whereas fewer immunolabeled cells were visible in the CO+HI group (Fig. 4D–F) and none were detected in CO+SHAM group (Fig. 4G–I). No significant differences were detected between the HI and CO+HI group with regard to glial activation and cell proliferation as assessed by S100β and Ki67 immunohistochemistry, respectively (data not shown).

### Effect of Carbon Monoxide on expression of apoptotic markers

**Figure 5:**
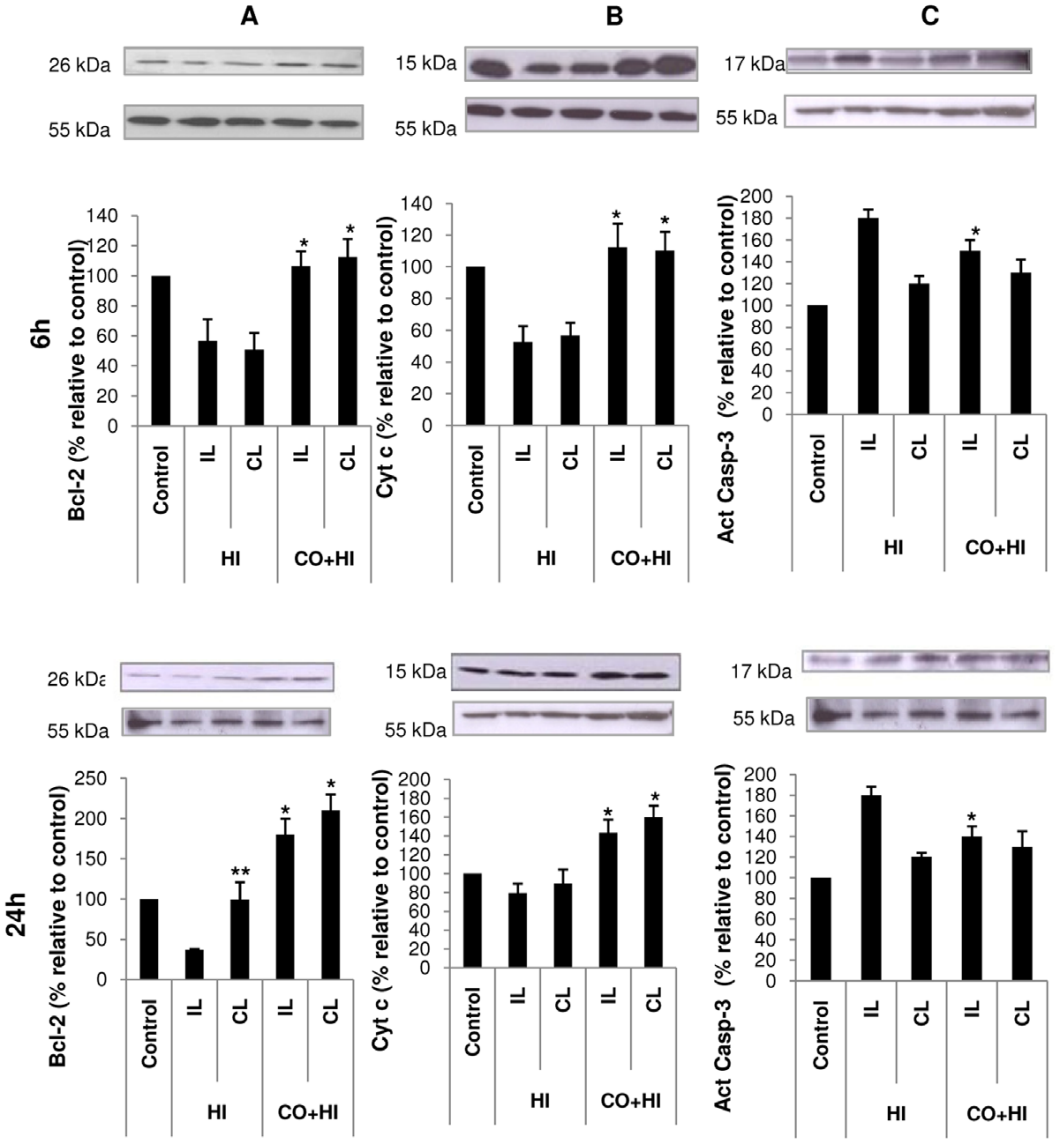
Effect of carbon monoxide on apoptotic markers in hippocampal extracts after 6 and 24 h of HI – protein expression and sub-cellular localization. Representative immunoblots (**upper panels**) and the corresponding quantifications, as relative percentages to the hippocampus from control rat pups (**lower panels**). (**A**) Bcl-2 expression in total hippocampal extracts. All values are mean ± SD (error bars), n = 3; *p<0.05 compared to HI group for the corresponding side and **p<0.05 compared to HI group ipsilateral hippocampus. (**B**) Cytochrome c levels in enriched mitochondrial fraction from hippocampus, which is an indirect way for measuring cytochrome c release. All values are mean ± SD (error bars), n = 3; *p<0.05 compared to HI group hippocampus for the corresponding side. (**C**) Caspase-3 activation in total extracts. All values are mean ± SD (error bars), n = 3; *p<0.05 compared to HI group for the ipsilateral hippocampus.

The results of immunoblots for apoptotic markes in total cellular extracts of ipsilateral (IL) and contralateral (CL) hippocampus or in mitochondria-enriched fractions are shown in Fig. 5. CO pre-treatment lead to an increased expression of anti-apoptotic protein Bcl-2 at 6 and 24 h in both hemispheres compared to the HI group. At 24 h, Bcl-2 expression among CO pre-treated pups was also higher than control pups (Fig. 5A). These data are in accordance with cell death prevention by CO in hippocampus and validate the *in vitro* results showing higher levels of Bcl-2 mRNA for CO-treated neurons (Fig. 2C). Therefore, CO appears to trigger tissue preconditioning and to stimulate cell survival, by changing gene expression, such as bcl-2. Cytochrome c levels in mitochondria-enriched fraction of hippocampus of the HI group were lower at 6 h than at 24 h after the insult relative to controls. At both 6 and 24 h after HI, CO pre-treated rat pups had higher levels of cytochrome c than the HI group in mitochondria-enriched fraction of hippocampus (Fig. 5B). Thus, CO prevented cytochrome c release from mitochondria, which can limit apoptosome formation and caspase-3 activation. Indeed, caspase-3 activation was less pronounced in IL hippocampal extracts from CO+HI group than from HI group at 6 and 24 h, while caspase-3 activation was similar in CL CO+HI and HI groups (Fig. 5C).

## Discussion

Two complementary approaches were applied to demonstrate the neuroprotective role of carbon monoxide: *in vitro* excitoxicity induction in primary culture of rat cerebellar neurons and an *in vivo* model of perinatal ischemia in rat pups. In primary neuronal cultures, 1 h of CO pre-treatment had a positive outcome on the survival of neurons against excitotoxic-induced cell death and increased expression of the anti-apoptotic gene *bcl-2* (Fig. 2). Likewise, prior exposure to CO significantly reduced the number of apoptotic profiles in the hippocampus (by 64%) 24 h following hypoxia-ischemia in rat pups (Fig. 3).

Apoptotic factors are up-regulated in early stages of brain development and play a major role in brain damage following perinatal ischemia because apoptosis is a key cellular process during CNS development [39], [40]. Therefore, apoptotic hallmarks were assessed *in vivo*. Indeed, CO pre-treatment improved the cellular anti-apoptotic machinery (Fig. 4 and 5). In accordance with the *in vitro* data, increased Bcl-2 expression was found in hippocampal extracts from CO-treated pups (Fig. 5A). Furthermore, increase in Bcl-2 expression activates many anti-apoptotic pathways and indicates that late preconditioning might be involved in CO neuroprotection [41]. Accordingly, previous studies have reported that protein synthesis is required for CO-conferred neuroprotection [25] and mitochondrial biogenesis stimulation is also involved in CO's apoptosis inhibition [35]. Thus, CO cytoprotection might be related to late preconditioning induction, although further studies are necessary. Nevertheless, early preconditioning responses cannot be excluded from CO's mode of action, since ROS signaling is involved in CO-induced cytoprotection [19], [26], [42], which presents new hypotheses for further studies based on cerebral cell anti-oxidant defense.

In hippocampus, higher cytochrome c levels in mitochondria were found with CO pre-treatment (Fig. 5B). This indicates a reduced release of cytochrome c from mitochondria, which can be related to the reported direct action of CO on mitochondrial membrane permeabilization [26]. Still, the increased cytochrome c sequestration in the mitochondria contributes to the lower levels of caspase-3 activation found in hippocampus from CO pre-treated pups (Fig. 5C). Furthermore, in hippocampal CA2, CA3 and DG regions, activated caspase-3 positive cell populations decreased when pups were exposed to CO before the hypoxic-ischemic insult (Fig. 4). An active role of CO in cerebral apoptosis prevention *in vivo* has been suggested by other authors: (i) HO-1 knockout mice exhibit a larger volume of tissue damage following injection of NMDA compared to wild type mice [43], and (ii) Zeynalov and colleagues report that low doses of exogenous CO protect against transient or permanent focal ischemia in adult mice [23], [24].

Perinatal HI can be partially predicted based on the presence of several risk factors: signals of distress during intrauterine life and hypoxic-ischemic insults at birth (Bonifacio et al. 2011). Also, preterm newborns represent a high-risk population for brain injury due to HI [44]. Therefore, preconditioning-based strategies can become potential therapies for perinatal HI, and CO is a promising candidate. Other preconditioning-based strategies have also been developed for perinatal cerebral ischemia. In piglets, 3 h of exposure to 8% oxygen prior to hypoxia/ischemia insult induced neuroprotection via up-regulation of hypoxia-inducible factor-1α (HIF-1α) and vascular endothelial factor (VEGF) [45]. Similarly, in adult mice, preconditioning by exposure to hypoxia and isoflurane presented a neuroprotective effect [46]. In an experimental model of HI similar to ours (left carotid artery ligation and 90 minutes of 8% O_2_), hydrogen has shown to be neuroprotective by blocking apoptosis [47]. Finally, lithium pre-treatment prevented apoptotic and autophagic neuronal cell death in the hippocampus following neonatal HI [48].

CO may be a useful therapeutic adjunct. In fact, other inhalation therapies have been introduced in clinics [49], to reduce toxicity resulting from the metabolization of administered drugs, since drug extraction occurs by exhalation. The therapeutic application of nitric oxide (NO) as vasodilator in several disease models and also in injured lungs of premature and newborn babies is now widely accepted [50]. NO is chemically similar to CO. However, unlike CO, NO reacts rapidly with molecular oxygen and produces peroxynitrite (ONOO^−^), which is highly reactive. Likewise, noble gases have also been studied for medical applications, especially xenon [51]. Recently, Ryang and co-workers [52] described the efficacy of argon in protecting rat brains in a model of transient middle cerebral artery occlusion. Taken all together, potential CO-inhaled based therapy has the added value of integrating two critical advantages: it is chemically inert compared to NO and is an endogenous molecule compared to noble gases [17].

To overcome a possible systemic CO toxic effect, the use of CO-releasing molecules (CORM's) has been proposed [53]. These small organic and organometallic compounds are able to deliver CO in a timely and tissue-specific manner. This permits a significant reduction in carboxyhaemoglobin formation and toxicity, which opens novel windows of opportunity for clinical applications. The biological activities of CORMs include cardioprotection in isolated perfused rat heart, protection in acute hepatic reperfusion injury in rats, endothelial cells protection during cold preservation, and injury impairment in the case of HI injury during kidney transplantation, among others [19], [54]. Very recently, CORM-3 was shown to modulate the inflammatory response and to reduce brain damage in an adult rat model of hemorrhagic stroke [55], indicating that CORM-3 can cross the blood brain barrier.

Altogether, *in vitro* and *in vivo* approaches have demonstrated that CO initiates a cascade of events that prevents neuronal apoptosis: (i) increase of Bcl-2 expression, (ii) prevention of cytochrome c release from mitochondria, (iii) inhibition of caspase-3 activation and (iv) decrease of chromatin condensation. CO administration induced late preconditioning and limited hippocampal neuronal cell death following cerebral perinatal ischemia. In conclusion, the biological neuroprotective role of CO, coupled with the possibility of using CORMs, opens avenues of further research and potential applications of CO-based therapies in cerebral ischemic models. Moreover, this innovative approach takes advantage of an endogenous molecule (CO) and intracellular pathways (preconditioning) for limiting neuronal cell death.

## Supporting Information

Figure S1 **Stereological measurement of apoptosis.** To estimate apoptosis, the hippocampal formation was traced in serial sections under 4× magnification, then counts were performed at 100× magnification (**A**); to allow unbiased sampling, counting sites were randomly selected by software at the end of tracing procedures (**B**). Each subsequent section was then superimposed and aligned to this reference slice to allow unbiased 3D-reconstruction of the hippocampus (**C–D**).
(PDF)
