# Supplementary material for: Preconditioning Triggered by Carbon Monoxide (CO) Provides Neuronal Protection Following Perinatal Hypoxia-Ischemia
Source: PLoS One. 2012 Aug 28;7(8):e42632. doi: 10.1371/journal.pone.0042632 (PMC3429477; doi:10.1371/journal.pone.0042632)
Supplement: Figure S1 — Stereological measurement of apoptosis. To estimate apoptosis, the hippocampal formation was traced in serial sections under 4× magnification, then counts were performed at 100× magnification (A); to allow unbiased sampling, counting sites were randomly selected by software at the end of tracing procedures (B). Each subsequent section was then superimposed and aligned to this reference slice to allow unbiased 3D-reconstruction of the hippocampus (C–D). (PDF) [file pone.0042632.s001.pdf]

FIG. S1

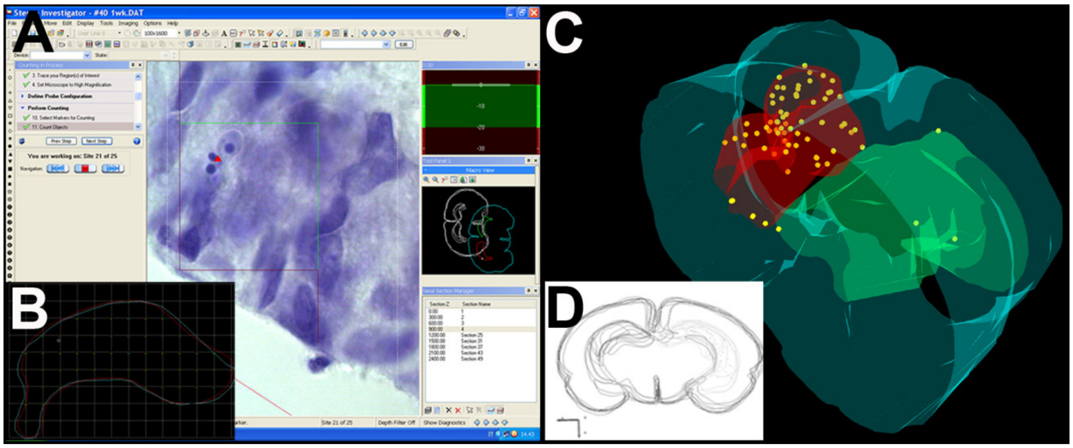

Fig. S1. Stereological measurement of apoptosis. To estimate apoptosis, the hippocampal formation was traced in serial sections under 4x magnification, then counts were performed at 100x magnification **(A);** to allow unbiased sampling, counting sites were randomly selected by software at the end of tracing procedures **(B).** Each subsequent section was then superimposed and aligned to this reference slice to allow unbiased 3D-reconstruction of the hippocampus **(C-D).**
